# Non-Canonical HIF2 Accumulation in Breast Cancer Cells

**DOI:** 10.3390/ijms27209306

**Published:** 2026-10-20

**Authors:** Karolina Kozal, Aleksandra Opitz, Monika Jarosiewicz, Michał Juszczak, Anna Krześlak

**Affiliations:** 1 Department of Cytobiochemistry, Faculty of Biology and Environmental Protection, University of Lodz, Pomorska 141/143, 90-236 Lodz, Poland; aleksandra.opitz@biol.uni.lodz.pl (A.O.); monika.jarosiewicz@biol.uni.lodz.pl (M.J.); 2 The BioMedChem Doctoral School of the University of Lodz and Lodz Institutes of the Polish Academy of Sciences, Jana Matejki 21/23, 90-237 Lodz, Poland; 3 Department of Molecular Genetics, Faculty of Biology and Environmental Protection, University of Lodz, Pomorska 141/143, 90-236 Lodz, Poland; michal.juszczak@biol.uni.lodz.pl

**Keywords:** hypoxia-inducible factor, breast cancer, metabolism, glycolytic efficiency, metabolic plasticity, Warburg effect

## Abstract

Metabolic reprogramming drives cancer aggressiveness. While hypoxia-inducible factor 1 (HIF1) is commonly associated with glycolysis regulation, the under-investigated HIF2 isoform may also modulate the metabolic profile of breast cancer. We investigated the role of non-canonical HIF2 activation in metabolic adaptation to nutrient availability by culturing MDA-MB-231 and MCF-7 cells under normoxia with varying glucose and glutamine concentrations. Using RNA interference, we downregulated HIF2 and analyzed HIF isoforms and metabolic targets (GLUT1, HK2, PKM2, PDK1) at the transcriptional and protein levels. We observed a new, non-canonical accumulation of HIF2 in MDA-MB-231 cells associated with glucose and glutamine restriction, a phenomenon absent in MCF-7 cells. In MDA-MB-231 cells, silencing *HIF2A* under standard conditions significantly decreased PDK1 mRNA expression. At the protein level, HIF2 downregulation altered metabolic targets in a context-dependent manner under nutrient restriction. Adding a hypoxia-mimetic (CoCl_2_) did not fully restore the protein expression patterns, suggesting that HIF2 accumulation may contribute to these context-dependent shifts. HIF2 downregulation decreased glycolytic efficiency under low-glucose conditions. Our findings suggest that HIF2 might modulate the metabolic plasticity of MDA-MB-231 cells through distinct changes in gene expression and post-translational mechanisms. This provides support for investigating the HIF2 isoform while studying breast cancer metabolism and considering it as a potential target for treating triple-negative breast cancer.

## 1. Introduction

Mammalian cells rely on exogenous energy sources, and the ability to utilize diverse nutrients is part of the metabolic plasticity vital for their growth and proliferation. Most cells prefer glucose; however, they can also resort to glutamine and fatty acids. The quantity and quality of consumed nutrients depend mostly on growth factor signaling, but are also regulated by intrinsic cellular mechanisms [1]. The demand for external nutrients is exceptionally high in cancer cells, whose abnormal rates of growth and proliferation are among the most fundamental hallmarks of cancer. The phenomenon of significantly increased glucose uptake and a high glycolytic rate, with glucose used to produce lactate even under normoxic conditions, known as the Warburg Effect, promotes cancer features. Reprogrammed metabolism serves purposes beyond meeting energetic requirements through ATP production, enabling the production of intermediates essential for the biosynthesis of various molecules, maintaining redox balance, stimulating cell growth and proliferation, and evading immune responses [2]. Because cancer cells depend heavily on glucose metabolism, its components have become attractive targets for new anticancer approaches, yet these approaches have not yielded significant breakthroughs [3]. Therapies targeting glycolysis may be insufficient due to adaptive mechanisms that respond to varying nutrient availability and energy levels [4]. Most cancer cells depend not only on exogenous glucose but also on glutamine, which is essential for producing purines, ATP, lactate, and fatty acids, and for maintaining redox balance [5,6,7].

A crucial element orchestrating metabolic reprogramming is activation of the hypoxia-inducible factor (HIF). It consists of two subunits: the oxygen-labile alpha subunit (which has three isoforms: 1α, 2α, and 3α) and the beta subunit, also called ARNT (Aryl Hydrocarbon Receptor Nuclear Translocator). HIF, consisting of the 1α or 2α subunit, will be referred to as the “HIF1” and “HIF2” isoforms, respectively. Under physiological oxygenation conditions, the alpha subunits have a half-life of approximately five minutes, after which they undergo proteasomal degradation. The process of alpha subunit disposal is regulated primarily by proteins belonging to the 2-oxoglutarate-dependent dioxygenase superfamily—prolyl hydroxylases (PHDs) [8]. Both isoforms share similarities in structure, regulation, and target genes. Still, they are canonically activated under different types of hypoxia, with HIF1 regulating the response to acute hypoxia and HIF2 involved in chronic hypoxia [8,9,10,11]. Solid tumors are often associated with hypoxic regions and decreased nutrient supply. Nevertheless, hypoxia is considered an unstable state [12] that increases heterogeneity in the metabolic profile [13]. Although insufficient oxygenation is a primary factor activating HIF, in cancers, its induction may occur through various non-canonical pathways [5,6,7,14,15,16,17,18,19], suggesting that its activity extends beyond adaptation to insufficient oxygenation.

HIF1 is the primary isoform associated with metabolic regulation. Its activity directly regulates the expression of key metabolic components, such as glucose transporters (mainly Glucose Transporter Type 1 GLUT1), glycolytic enzymes (phosphoglycerate kinase; aldolase; hexokinases; pyruvate kinase), pyruvate dehydrogenase kinases (especially PDK1, which increases the Warburg Effect by shunting pyruvate away from the tricarboxylic acid cycle, effectively decreasing oxidative phosphorylation (OXPHOS)), and glutamine transporters. Additionally, it participates in the Protein Kinase B (Akt) signaling pathway, which increases glucose transporter translocation and the activity of the first glycolysis-limiting enzyme, hexokinase II. HIF1 activity is generally recognized as closely associated with cancer metabolic profiles, and its inhibition could counteract this reprogramming [1,5,7,19,20]. However, the often under-investigated HIF2 isoform may also contribute to cancer metabolic plasticity, particularly under hypoxic conditions, as HIF1 levels decrease over time while HIF2 levels remain stable [21]. Although often considered redundant, HIF1 and HIF2 levels are not correlated in breast cancers, suggesting distinct mechanisms of their stabilization and activation [22]. As the role of the HIF2 isoform in regulating various features of breast cancer is gaining recognition [23,24,25,26], it is important to consider and distinguish between the two isoforms. The roles of the hypoxia-inducible factor HIF1 and HIF2 isoforms in breast cancer biology are presented and discussed in more detail in the review [27].

In our study, we investigated how glucose and glutamine availability affects non-canonical activation of HIF1 and HIF2 in two widely studied breast cancer cell lines (MCF-7, MDA-MB-231) cultured under normoxia, and how varying nutrient availability, coupled with modulation of HIF2 levels, affects the expression of metabolism-associated genes.

## 2. Results

### 2.1. The Effects of Glucose and Glutamine Availability on the Expression and Accumulation of Hypoxia-Inducible Factor Isoforms

To investigate how nutrient availability affects the expression of hypoxia-inducible factor isoforms, we analyzed the effects of medium and low glucose (5 mM vs. 0.5 mM) and standard and medium glutamine (2 mM vs. 1 mM) on *HIF1A* and *HIF2A* mRNA levels. Cobalt chloride was included in the experimental setup as a hypoxia-mimetic agent, inhibiting the degradation of the α-subunit of hypoxia-inducible factor and preserving its level. The expression analysis results are shown in Figure 1.

The highest level of *HIF1A* mRNA in MDA-MB-231 cells was observed under low-glucose conditions, with a pronounced decrease under low-glucose and glutamine conditions. For the *HIF2A*, the highest upregulation under medium glucose was not followed by a similar decrease under low-glucose and glutamine conditions, suggesting that, in that cell line, regulation of *HIF2A* mRNA levels might be less sensitive to limited glutamine availability. The pattern of changes in mRNA expression for both HIF isoforms persists between cells cultured in the absence and in the presence of 100 µM CoCl_2_, indicating that the observed effects are independent of post-translational stabilization. In MCF-7 cells, changes in HIF isoform expression patterns exhibit opposite tendencies with respect to glucose availability. The highest *HIF1A* upregulation was observed under medium-glucose conditions; however, as in MDA-MB-231 cells, expression declined significantly when glutamine availability decreased. For *HIF2A*, isoform expression was the highest in cells cultured in low-glucose conditions, with downregulation under medium-glutamine conditions. The expression levels of both HIF isoforms were higher in MCF-7 cells than in MDA-MB-231 cells.

As previously reported, mRNA levels of hypoxia-inducible factors and observed protein levels often diverge due to post-translational regulation of their stabilization. To determine the levels of accumulated hypoxia-inducible factors, we used Western blotting. The densitometric results for bands corresponding to the HIF1 and HIF2 isoforms are presented in Figure 2. In MDA-MB-231 cells, the HIF1 isoform remained almost undetectable without CoCl_2_ stabilization, except in cells cultured under low-glucose and glutamine conditions. The highest observed level of HIF1 under additional stabilization was in medium-glutamine conditions, with a more prominent response in medium-glucose conditions, suggesting that glutamine might be limiting for that isoform of hypoxia-inducible factor. HIF2 isoform was detectable across all nutrient availability conditions, even without cobalt chloride stabilization. In cells cultured without hypoxia-mimetic addition, HIF2 accumulation appeared most prominent under medium-glutamine conditions, with a more pronounced effect under low-glucose conditions. CoCl_2_ addition induced the highest detected accumulation at standard glutamine and medium glucose concentrations. In MCF-7 cells, both isoforms were almost undetectable across all conditions without additional stabilization. In cells cultured with cobalt chloride, HIF1 accumulation was most prominent under medium-glutamine conditions, especially at decreased glucose concentrations. For the HIF2 isoform, the maximum observed accumulation was observed under medium-glucose and standard-glutamine conditions. Levels observed in medium-glutamine matched trends in HIF1 isoform patterns. Overall, despite higher mRNA levels of both HIF isoforms in Figure 1, MCF-7 cells showed lower accumulated levels of HIF1 and HIF2 proteins than MDA-MB-231 cells, as observed in Figure 2.

### 2.2. The Effects of HIF2A Silencing and Glucose and Glutamine Availability on the Expression of Proteins Associated with Metabolism

Because we observed robust HIF2 accumulation exclusively in the MDA-MB2-31 cell line, we selected it to investigate downstream molecular changes following HIF2 downregulation. We employed *HIF2A* silencing in breast cancer cells cultured at various glucose and glutamine concentrations. Because a limited glutamine concentration markedly altered both hypoxia-inducible factor isoforms, we further reduced it. To evaluate functional downstream effects of HIF2 downregulation, subsequent siRNA experiments were compared to standard 25 mM glucose conditions as unstressed baseline. MDA-MB-231 cells were cultured in standard (25 mM glucose, 2 mM glutamine), low-glucose (0.5 mM glucose, 2 mM glutamine), or low-glucose and glutamine (0.5 mM glucose, 0.2 mM glutamine) conditions for 48 h under normoxia. Cells were treated with siRNA targeting *HIF2A* or scrambled siRNA.

To validate the efficiency of *HIF2A* silencing, we evaluated *HIF1A* and *HIF2A* expression at the transcriptional level under standard conditions (Figure 3A). Transfection with HIF2-targeting siRNA significantly reduced *HIF2A* mRNA levels compared with the scrambled control. Importantly, the knockdown was specific to the HIF2 isoform, as *HIF1A* mRNA levels remained unaffected. We evaluated the effects of HIF2 downregulation under nutrient-limited conditions, which was shown to enhance HIF2 protein accumulation in MDA-MB-231 cells, using additional CoCl_2_ treatment as a stabilization benchmark (Figure 3B). While cells treated with scrambled siRNA showed notable HIF2 accumulation under standard conditions (S) and increased band intensity under nutrient-deprived conditions (↓Glc, ↓Glc, Q), silencing *HIF2A* reduced HIF2 accumulation despite the hypoxia-mimetic, confirming successful downregulation across conditions.

To assess the effects of *HIF2A* silencing in breast cancer cells cultured under various glucose and glutamine availability on the proteins associated with metabolism, the following targets were selected: glucose transporter type 1 (GLUT1, SLC2A1 gene), hexokinase 2 (HK2), pyruvate kinase M2 (PKM2), and pyruvate dehydrogenase kinase 1 (PDK1). MDA-MB-231 cells were cultured in standard S (25 mM glucose, 2 mM glutamine), low-glucose ↓Glc (0.5 mM glucose, 2 mM glutamine), or low-glucose and glutamine ↓Glc, Q (0.5 mM glucose, 0.2 mM glutamine) conditions for 48 h under normoxia, and treated with siRNA targeting *HIF2A* or scrambled siRNA.

To investigate the effects of *HIF2A* silencing on metabolic targets, their expression was first evaluated at the transcriptional level (Figure 4A). HIF2 downregulation under standard conditions was associated with a significant decrease in PDK1 mRNA levels, whereas GLUT1, HK2, and PKM2 mRNA levels remained unchanged. Results of the Western blot analysis shown in Figure 4B indicate that silencing *HIF2A* was associated with a slight decrease in the levels of all metabolic proteins, except GLUT1. However, the addition of cobalt chloride, which stabilizes the glycolysis-associated HIF1 isoform, did not fully restore their levels compared to those observed in cells treated with scrambled siRNA. Cells cultured under low-glucose (↓Glc) conditions showed increased levels of GLUT1 and PDK1, and of GLUT1, HK2, and PKM2 under the same conditions with additional hypoxia-mimetic treatment. This suggests that observed trends in changes in metabolism-associated proteins cannot be attributed solely to HIF1 upregulation following glucose restriction, as the patterns of change should be similar in cells cultured in the absence and presence of cobalt chloride. Similar associations were observed in cells cultured under low-glucose and glutamine conditions (↓Glc, Q). Still, the changes in GLUT1 and PDK1 patterns were distinct between cells treated with CoCl_2_ and untreated *HIF2A*-downregulated MDA-MB-231 cells, suggesting that HIF2 accumulation might be associated with these proteins under nutrient-limited conditions. Changes in the levels of metabolism-associated proteins following *HIF2A* silencing in MDA-MB-231 cells cultured at various glucose and glutamine concentrations are illustrated in Figure 4. To determine how *HIF2A* silencing influences the stoichiometric conversion of glucose to lactate in MDA-MB-231 cells under nutrient-deprived conditions, we measured glucose consumption and lactate production to calculate glycolytic efficiency. The most prominent differences were observed in glucose consumption, which notably dropped coincidentally with HIF2-downregulation in low-glucose conditions (Figure 5A), aligning with a subsequent drop in glycolytic efficiency under the same nutrient restriction compared to the scrambled control (Figure 5B). No significant changes in glycolytic efficiency were observed under combined glucose and glutamine restriction.

## 3. Discussion

Hypoxia-inducible factor activity is primarily regulated through post-translational control, in which the HIFα subunit undergoes oxygen-dependent degradation or is stabilized under hypoxic conditions, often resulting in discrepancies among observed mRNA levels, protein levels, and HIF activity [16,28]. This discrepancy is consistent with previous observations indicating that HIF signaling is regulated at multiple levels. Filippi et al. demonstrated that HIF1 recruitment to the target promoter may occur despite impaired transcriptional activity, highlighting that HIF stabilization alone does not necessarily reflect functional transcriptional changes [29]. Consistent with findings from previous research, we observed distinct patterns between changes in hypoxia-inducible factor transcripts and accumulated proteins. While MCF-7 cells showed generally higher mRNA levels for both isoforms, this did not reflect protein abundance. In contrast, MDA-MB-231 cells exhibited greater HIF accumulation. Glucose levels markedly affected mRNA expression; however, protein-level analysis revealed that additional glutamine restriction had a greater impact on HIF levels. The HIF1 isoform remained almost undetectable without CoCl_2_ stabilization in both cell lines, whereas the HIF2 isoform was detectable without additional stabilization under glutamine-limited conditions in MDA-MB-231 cells. These findings reveal a new, non-canonical accumulation of HIF2 in response to glucose and glutamine limitation in MDA-MB-231 cells, which contrasts with previous reports of the absence of the HIF2 isoform in triple-negative breast cancer (TNBC) cells [16]. MDA-MB-231 cells exhibit the highest proliferation rate and invasive potential among triple-negative breast cancer cell lines. They are considered to have a highly glycolytic phenotype associated with high lactate production [30], and endogenous lactate has been reported to stimulate time-dependent overexpression of *HIF1A* [31]. Still, no such observation was made for the HIF2 isoform so far. Impaired PHD activity, associated with L-glutamate secretion, has been reported to stabilize HIF protein levels. Such a phenomenon was observed in glutamine-dependent TNBC; however, only for the HIF1 isoform [16], and it could not explain the increased accumulation in conditions of limited glutamine, which serves as a precursor for glutamate. In fact, abundant glutamine availability has been shown to induce normoxic HIF1 accumulation in MDA-MB-231 cells [32], so observing HIF2 accumulation under glutamine-deprived conditions might suggest a compensatory metabolic shift required for adaptation to nutrient scarcity. Moreover, loss of HIF1 did not significantly affect the metabolic phenotype of cancer cells [33]. In our previous work, we demonstrated a negative correlation between HIF2 and O-GlcNAcylation level in breast cancer. As a type of glycosylation, O-GlcNAcylation remains strictly dependent on the availability of glucose and glutamine via the hexosamine biosynthetic pathway (HBP). We hypothesize that reduced O-GlcNAcylation may contribute to the mechanism underlying HIF2 accumulation under nutrient-deprived conditions [34].

Meanwhile, MDA-MB-231 cells remain more resilient to starvation, despite being highly glycolytic and insensitive to insulin and glycolytic inhibitors [35,36,37]. We hypothesize that this non-canonical accumulation of HIF2 might contribute to adaptation to nutrient stress by modulating the metabolic plasticity of these breast cancer cells. Under standard conditions, silencing *HIF2A* did not trigger a compensatory upregulation of *HIF1A*, indicating a lack of immediate transcriptional crosstalk between HIF isoforms. siRNA-mediated silencing successfully decreased HIF2 accumulation under standard and low-nutrient conditions, even in the presence of a hypoxia-mimetic, but the increase in band intensity under low-glucose conditions is noteworthy. Coupling it with the observation of HIF2 accumulation under glucose and glutamine restriction in normoxia suggests a potential role in adaptation to limited nutrient availability.

The primary mechanism by which hypoxia-inducible factor regulates the metabolic profile of breast cancers is transcriptional activation of genes encoding key components of glucose metabolism. Although this role is usually attributed to the HIF1 isoform, we wanted to investigate whether HIF2 activity under low-glucose and low-glutamine conditions would affect the expression of metabolic genes. For that, we chose the following targets. The first discovered glucose transporter, GLUT1 (SLC2A1) [7], a predominantly overexpressed isoform in breast cancer, is associated with poorer prognosis and is upregulated in both MCF-7 and MDA-MB-231 cell lines [36]. Canonically, it is regulated by the HIF1 isoform but has also been reported to be controlled by the HIF2 isoform [17]. Hexokinase II (HK2), the first enzyme of the glycolytic pathway, also serves as the first glycolytic checkpoint and is transcriptionally regulated by the HIF1 isoform [19]. It was shown that increased HK2 activity might be crucial for surviving glucose-limited conditions, especially in triple-negative breast cancer cells, and is associated with poor prognosis [38]. Pyruvate kinase M2 (PKM2), involved in the final step and the checkpoint of glycolysis, has lower catalytic activity than the M1 isoform, which, while slowing glycolysis, enables the redirection of glucose intermediates into alternative pathways, including the production of UDP-glucose required for O-GlcNAcylation [39]. It was also shown to transactivate HIF1 under hypoxic conditions [18]. Pyruvate dehydrogenase kinase 1 (PDK1), whose overexpression in breast cancer has been linked to HIF1 upregulation in MDA-MB-231 cells [20,36], phosphorylates a subunit of the pyruvate dehydrogenase complex, thereby shunting pyruvate away from the tricarboxylic acid cycle and decreasing its availability for oxidative phosphorylation. PDK1 has been reported to stabilize HIF1 levels [40], but its relationship to HIF2 has not been investigated. Decreased HIF1 does not always lead to downregulation of genes associated with metabolism [18,41]. It could result from HIF-independent metabolic regulation via mTOR signaling [5], activity of Phosphofructokinase-1 (PFK1) [33], or activity of the HIF antisense long non-coding RNA (HIFAL), which might help sustain HIF1 transcriptional activity despite reduced stabilization [18]. However, given the observation that silencing HIF through ARNT (hypoxia-inducible factor beta subunit), required for creating an active heterodimer for both HIF1 and HIF2 isoforms, resulted in downregulation of PDK1 in MDA-MB-231 cells cultured in normoxic conditions [41], the HIF2 isoform is also likely involved in the modulation of metabolic gene expression.

We observed distinct modulation of metabolic targets in MDA-MB-231 cells at the transcriptional and protein levels. Under standard conditions, *HIF2A* silencing was associated with a significant decrease in PDK1 mRNA levels, whereas GLUT1, HK2, and PKM2 levels remained unchanged. However, at the protein level, HIF2 downregulation was associated with a slight downward trend in all evaluated proteins except GLUT1. For GLUT1 and PDK1, distinct expression patterns across nutrient availability in cells with downregulated *HIF2A*, with or without a hypoxia-mimetic, indicate that the observed trends cannot be attributed solely to HIF1 upregulation following glucose limitation. Given that cobalt chloride addition failed to fully restore these expression patterns, it suggests that HIF2 accumulation might contribute to the context-dependent modulation of these proteins under nutrient-limited conditions. These findings indicate that, despite the common association of the HIF1 isoform with the regulation of metabolic genes, HIF2 might also contribute to their regulation, either through transcriptional modulation, as with PDK1, or through context-dependent regulation of protein abundance under basal or nutrient-deprived conditions in the MDA-MB-231 triple-negative breast cancer model. Observing this HIF2-mediated modulation of PDK1 expression is particularly significant, as PDK1 plays a crucial role in metabolic reprogramming that drives metastatic potential in breast cancer cells [42]. In vivo results strongly support the idea that HIF1 and HIF2 isoforms have distinct roles in breast cancer metabolism, showing that in MDA-MB-231-derived xenografts, silencing *HIF1A* and *HIF2A* results in highly divergent metabolic profiles [43].

Glucose consumption is directly correlated with the aggressiveness of breast cancer cells [36], and glycolytic efficiency is linked to the proliferation rate and therapy resistance [4]. We observed that under glucose-limited conditions, the lactate-to-glucose ratio in MDA-MB-231 cells exceeded the theoretical stoichiometric maximum of 2.0, which requires the input of alternative carbon sources. This phenomenon may result from sustained lactate production despite limited glucose consumption under the experimental conditions. MDA-MB-231 cells are highly glycolytic compared with other breast cancer cell lines [6,7,30,31,35], and their glutamine dependence means they can produce a significant amount of this additional lactate through glutaminolysis [7,44]. However, a notable drop after *HIF2A* silencing in glucose-limited conditions suggests that this isoform might play a role in sustaining the metabolic phenotype of breast cancer cells under nutrient restriction. We hypothesize that PDK1 mediates this association. MDA-MB-231 cells have higher PDK1 mRNA levels than MCF-7 cells [16], and PDK1 inhibition redirects cellular metabolism toward OXPHOS, lowering glycolytic efficiency and proliferation [4].

Since the lack of hormone receptors results in limited therapeutic options in triple-negative breast cancer, and this subtype is associated with a higher probability of metastasis and a higher death rate [45], it is especially important to elucidate the molecular features that enhance progression and increased aggressiveness. In TNBC, the high glycolytic flux observed even in the presence of oxygen, recognized as the Warburg Effect, drives progression and therapy resistance. This metabolic feature is primarily mediated by glycolysis-associated enzymes, including hexokinase (HK), phosphofructokinase (PFK), pyruvate kinase M (PKM), and lactate dehydrogenase (LDH) [46]. Shifts in the expression and activity of these enzymes not only promote the production and accumulation of glycolytic intermediates needed to meet anabolic demands, but also drive radioresistance in TNBC [47]. Radiotherapy stabilizes hypoxia-inducible factor (HIF), which may create a feedback loop that increases HIF-mediated metabolic reprogramming and protects cells from reactive oxygen species by activating the Pentose Phosphate Pathway (PPP) in parallel with glycolysis [48]. High glycolytic efficiency also drives higher proliferation of breast cancer cells through enhanced PPP [4]. A better understanding of the role of hypoxia-inducible factor isoforms in shaping glycolytic flux in triple-negative breast cancer might thereby provide information crucial for increasing sensitivity to radiotherapy.

We observed a link between non-canonical HIF2 accumulation and the metabolic plasticity of MDA-MB-231 cells, but we acknowledge the study’s limitations. While we used cobalt chloride to establish a baseline for HIF stabilization, it is associated with off-target effects, including reactive oxygen species (ROS) generation and mitochondrial alterations. Our data show an association between HIF2 accumulation and metabolic proteins, but direct transcriptional regulation requires further investigation. Given our findings in the MDA-MB-231 model, it would be valuable to test other TNBC cell lines to determine whether this phenomenon is specific to this cell line or the breast cancer subtype. We conducted in vitro experiments in a 2D model, which often does not reflect the full picture, especially given that hypoxia, which naturally occurs after a tumor exceeds a certain size, might increase heterogeneity in the metabolic profile [13]. Findings should be further validated in 3D and in vivo models of breast cancer. Although we have linked a non-canonical HIF2 accumulation to the glutamine restriction, the exact mechanism remains to be elucidated. Future investigations will explore the molecular interplay between O-GlcNAcylation, a nutrient sensor sensitive to glutamine availability, and HIF2 accumulation. It would be beneficial to use not only *HIF2A* knockdown but also inhibitors that directly bind HIF2 or ARNT, which are gaining interest [49], with the HIF2 inhibitor belzutifan (MK-6482) already planned for testing as a combination therapy in metastatic breast cancer (ClinicalTrials.gov ID: NCT06428396).

If the novel, non-canonical HIF2 accumulation were to prove to be specific for the triple-negative breast cancer subtype, it would contextualize HIF2 inhibitors in treatment strategies. Since HIF1 lacks direct inhibitors [27], drugs targeting it indirectly are associated with a higher risk of off-target effects. Moreover, the HIF1 isoform is much more broadly distributed throughout the body, increasing the likelihood of adverse effects. The HIF2 isoform remains more tissue-specific and can be targeted directly by belzutifan. Although our study is too preliminary to establish a mechanistic link between nutrient restriction and HIF2 accumulation, it supports including the HIF2 isoform when investigating hypoxia-inducible factor in the context of breast cancer metabolism.

## 4. Materials and Methods

### 4.1. Cell Culture and Treatment

Human breast cancer lines MCF-7 and MDA-MB-231 were obtained from the American Type Culture Collection (Manassas, VA, USA) and stored in liquid nitrogen until used. Both cell lines were cultured in Dulbecco’s Modified Eagle Medium (DMEM) with 10% fetal bovine serum (FBS), 2 mM L-glutamine, and 25 mM glucose. Cells were maintained as monolayer cultures in a humidified 5% CO_2_ and ~21% O_2_ atmosphere at 37 °C. Cells were subcultured every 3–5 days. Prior to experiments, cells were tested and confirmed to be free of mycoplasma contamination using Mycolor One-Step Mycoplasma Detector (Vazyme, Nanjing, China). In experiments, cells were cultured for 48 h in DMEM with 10% FBS, and concentrations of glucose and glutamine presented in Table 1.

**Table 1:** Concentration of glucose and glutamine in experimental medium.

Conditions	Glucose (mM)	Glutamine (mM)
Standard	25	2
Low glucose	0.5	2
Medium glucose	5	2
Low glucose and medium glutamine	0.5	1
Medium glucose and medium glutamine	5	1
Low glucose and low glutamine	0.5	0.2

For the additional HIF stabilization, cells cultured under certain conditions were treated with 100 µM CoCl_2_. Cobalt chloride was used to prevent oxygen-dependent degradation of the HIF-α subunit by inhibiting PHD activity [50], and its concentration was chosen based on the induction of hypoxia-response gene expression and cell viability, as validated in pilot experiments [51].

### 4.2. RNA Interference

*HIF2A* expression was downregulated using predesigned siRNA purchased from Life Technologies (Carlsbad, CA, USA) (s4699) and transfected with Lipofectamine^®^ 2000 Transfection Reagent (Invitrogen, Carlsbad, CA, USA) according to the manufacturer’s instructions. The optimal siRNA concentration of 30 nmol/L was established in the pilot experiment, as it sufficiently silenced *HIF2A*, as confirmed by PCR and Western blot, while avoiding off-target effects. Results from *HIF2A* silencing were compared with the control, cultured under matching conditions and treated with Silencer™ Negative Control No. 1 siRNA from Ambion™ (Carlsbad, CA, USA) at the same siRNA concentration. Cells were incubated for 48 h with a change to siRNA-free medium after 24 h.

### 4.3. Real-Time PCR

Total RNA was extracted from cells using Fenozol reagent (A&A Biotechnology) according to the manufacturer’s instructions, followed by first-strand cDNA synthesis using High-Capacity cDNA Reverse Transcription Kit (Life Technologies, Carlsbad, CA, USA), following the recommended protocol. Real-time cDNA amplification was performed using specific primers and SYBR Green. Specificity was confirmed during gradient PCR. Post-qPCR DNA samples were analyzed by 2% agarose gel electrophoresis in 1× TAE buffer; 10 μL of the DNA sample was mixed with 2 μL of DNA Gel Loading Dye (6×). Electrophoresis was run for 60 min at 100 V in 1× TAE buffer. Products were identified by using 4 μL of 100 bp DNA ladder (Perfect 100 bp DNA ladder, Eurx, Gdansk, Poland). Samples were then stained with ethidium bromide, visualized under UV light (302 nm), scanned with a CCD camera, and analyzed with GeneTools software version 4.3.9.0 (Syngene, Cambridge, UK). The qPCR was performed using LightCycler^®^ 480 Systems (Roche, Basel, Switzerland)). Relative transcript levels were calculated using the 2^−ΔΔCt^ method, with *HPRT1* and *PUM1* levels used as internal controls. Results were normalized to both references and presented as an arithmetic mean. Primer sequences used for gene amplification, gradient PCR results, and gel images from electrophoretic validation are provided in the Supplementary Materials.

### 4.4. Western Blot

Cells were harvested with trypsin and lysed with RIPA buffer (50 mM Tris-HCl pH 8, 150 mM NaCl, 0.5% sodium deoxycholate, 1% Nonidet P-40, 0.1% SDS, 1 mM EDTA, 1 mM PMSF) and sonicated (amplitude 25% for 30 s). Protein concentration in the samples was determined using the Lowry method. 30 µg of each sample was loaded, resolved by 8% SDS-PAGE at a constant current of 50 mA, and transferred overnight onto a 0.45 µm PVDF transfer membrane at a constant voltage of 50V. Electrophoresis and transfer efficiency were evaluated using Ponceau S staining, and the membranes were blocked with 5% non-fat milk. HIF1 and HIF2 were detected on separate membranes to avoid errors due to identical molecular weight, and blots were processed in 2 sets in parallel. The arithmetic mean of two references, Pumilio 1 (PUM1) and tubulin beta 3 class III (TUBB3), was used to normalize the results. Antibodies used for immunodetection are listed in the Supplementary Materials. Integrated optical density was performed using ImageJ software (version: 1.53k). Results are reported as relative protein levels compared with the mean expression of references, or as fold change compared with the control in *HIF2A*-downregulated samples. Full immunodetection images are provided in the Supplementary Materials.

### 4.5. Glucose Consumption and Lactate Production Assays

Both glucose consumption and lactate production were measured in spent medium samples collected at the 0 h, 24 h, and 48 h time points. After pooling triplicate samples, the samples were centrifuged twice to remove cellular debris (1000× *g*, 13,000× *g*) and stored at −80 °C until assays were performed. Cells were harvested using trypsin and lysed with RIPA buffer (50 mM Tris-HCl pH 8, 150 mM NaCl, 0.5% sodium deoxycholate, 1% Nonidet P-40, 0.1% SDS, 1 mM EDTA, 1 mM PMSF) and sonicated (amplitude 25% for 30 s). Protein concentration in the samples was determined using the Lowry method. Upon conducting assays, samples were diluted, and the Glucose Assay Kit ab65333 and L-Lactate Assay Kit ab65331 (Abcam, Cambridge, UK) were performed according to the manufacturer’s instructions. Glucose consumption and lactate production were calculated from the colorimetric readouts (optical densities at 570 nm and 450 nm, respectively) and normalized to the dilution factor, reduced media volumes after sampling, and the total protein mass of pooled triplicate experiments. Glycolytic efficiency was calculated as the stoichiometric ratio of lactate production to glucose consumption [4].

### 4.6. Statistical Analysis

Analyses were performed with STATISTICA 10 (StatSoft, Kraków, Poland) and GraphPad Prism 11 (Dotmatics, Boston, MA, USA). Data were evaluated for outliers prior to analysis. The ROUT method (Q = 1%) was utilized as the primary method, but groups with restricted sample sizes (N = 3) were analyzed with the Grubbs’ test (α = 0.05). Groups were compared using the Mann–Whitney or the Kruskal–Wallis test, followed by Dunn’s post hoc test for multiple comparisons. Results are presented as medians. A *p*-value < 0.05 was considered to indicate a statistically significant difference. A full statistical description, including group sizes, the test used, and exact results is provided in the Supplementary Materials.

## 5. Conclusions

Our study reveals a non-canonical accumulation of HIF2 in response to glucose and glutamine restriction in the triple-negative breast cancer cell line MDA-MB-231. Observing this phenomenon suggests a possible mechanism for cellular adaptation to metabolic stress. HIF2 accumulation appears to be associated with adaptive shifts through both changes in PDK1 expression and the context-dependent changes in the abundance of other metabolic proteins such as GLUT1. These findings carry clinical implications. The role of HIF2 in maintaining metabolic plasticity remains under-investigated but may enable highly glycolytic cells, such as the MDA-MB-231 TNBC model, to adapt to nutrient-deprived conditions, thereby increasing their aggressiveness. HIF2-associated adaptability could partially explain why limiting glycolysis or inhibiting HIF1 sometimes fails to affect the growth, proliferation, or aggressiveness of BC cells. Targeting HIF2 might provide a useful addition in breast cancer therapy, especially for aggressive, nutrient-resilient phenotypes common in the triple-negative breast cancer subtype.

## Figures and Tables

**Figure 1:**
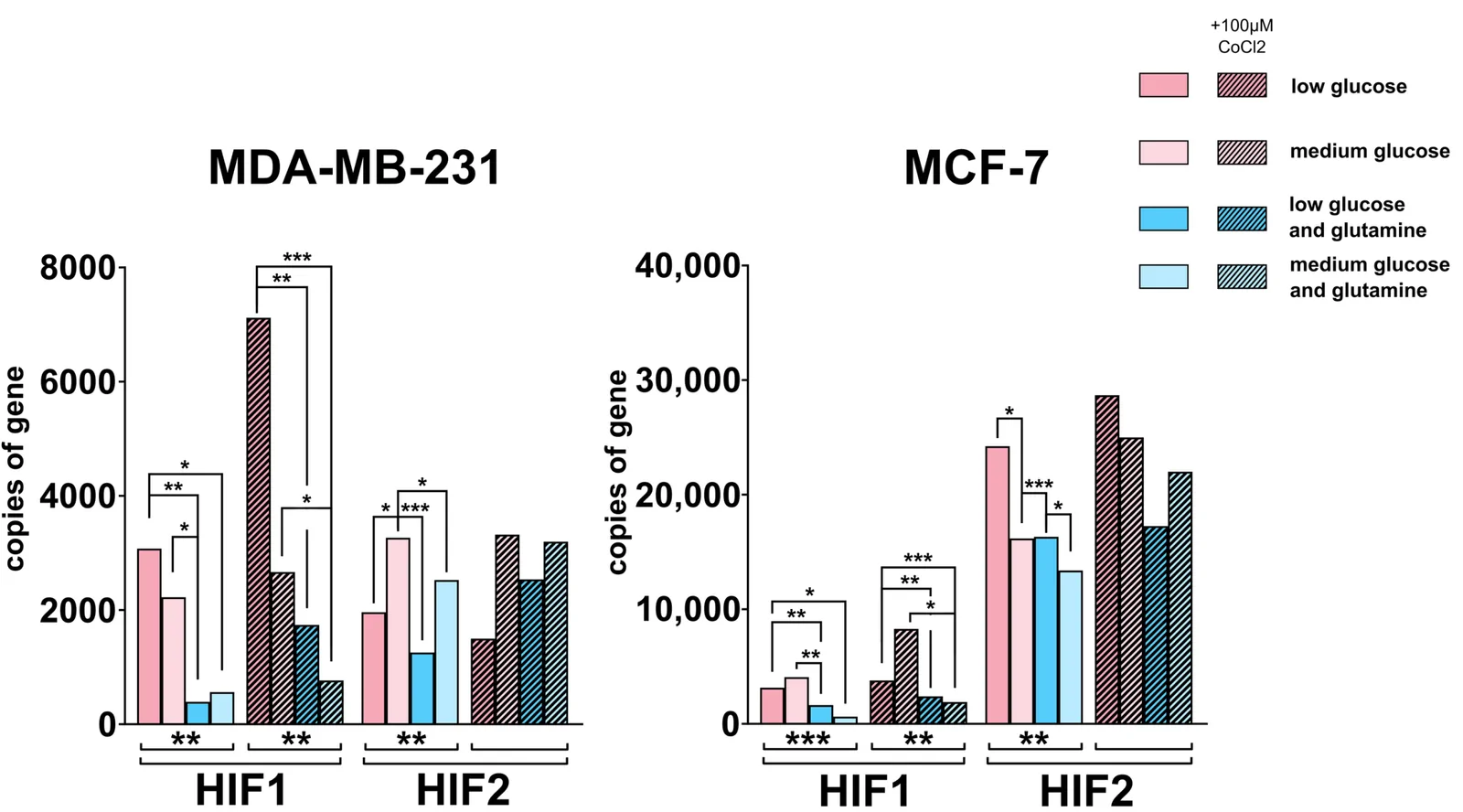
The effects of different nutrient availability on HIF1 and HIF2 mRNA levels. MDA-MB-231 and MCF-7 cells were cultured for 48 h in low glucose (0.5 mM glucose, 2 mM glutamine), medium glucose (5 mM glucose, 2mM glutamine), low glucose and glutamine (0.5 mM glucose, 1 mM glutamine), or medium glucose and glutamine (5 mM glucose, 1 mM glutamine), under normoxia, with 100 µM CoCl_2_ indicated by the striped pattern. Bars show the medians of the number of gene copies obtained during PCR, normalized to the PUM1 and HPRT1 housekeeping genes using the 2^−ΔΔCt^ method. The Kruskal–Wallis test was used (indicated by the asterisks below the respective group), followed by Dunn’s post hoc test for multiple comparisons (indicated by asterisks above brackets connecting specific groups). Established statistical significance (α = 0.05, * *p* ≤ 0.01, ** *p* ≤ 0.001, *** *p* ≤ 0.0001) is displayed only for cells cultured in the same conditions regarding the addition of cobalt chloride. A full description of the statistical analysis, including the sample sizes and tests used, is provided in the Supplementary Materials.

**Figure 2:**
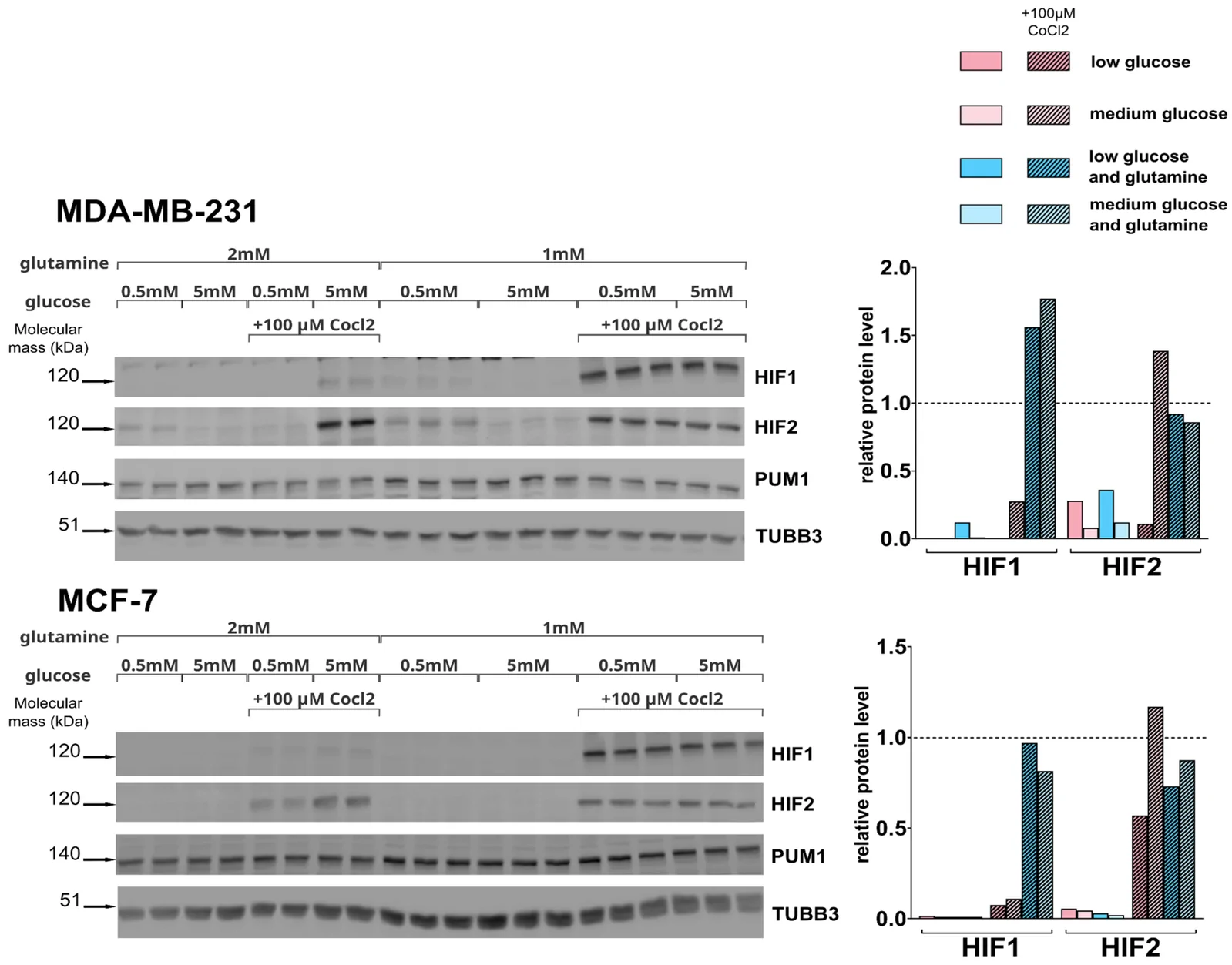
Effects of different nutrient availability on HIF1 and HIF2 protein levels. MDA-MB-231 and MCF-7 cells were cultured for 48 h in low glucose (0.5 mM glucose, 2 mM glutamine), medium glucose (5 mM glucose, 2mM glutamine), low glucose and glutamine (0.5 mM glucose, 1 mM glutamine), or medium glucose and glutamine (5 mM glucose, 1 mM glutamine), under normoxia, with 100 µM CoCl_2_ added as indicated by the striped pattern. The Western blot images are accompanied by graphs showing the results of integrated optical density measurements. Bars show the medians of relative protein levels from Western blots, normalized to the PUM1 and TUBB3 reference proteins, with the dashed line at 1.0 as the baseline, corresponding to the average of the reference proteins. Due to the sample size constraints, data are presented as descriptive observations, and formal statistical analysis was omitted. HIF1 and HIF2 were detected and processed on two parallel blots; the figure shows representative images. Full immunodetection images are provided in the Supplementary Materials.

**Figure 3:**
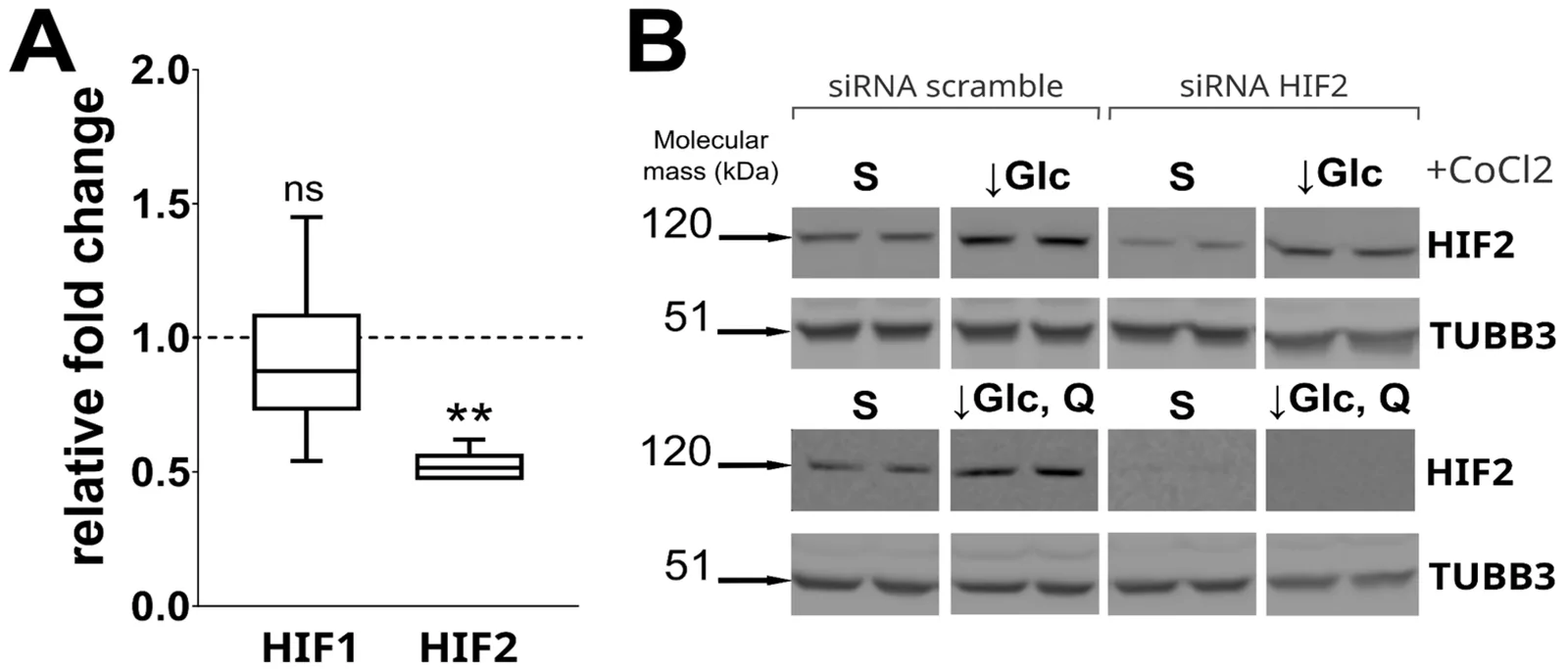
The effects of *HIF2A* silencing under different glucose and glutamine availability on the expression and accumulation of hypoxia-inducible factor isoforms. MDA-MB-231 cells were cultured for 48 h in standard S (25 mM glucose, 2 mM glutamine), low-glucose ↓Glc (0.5 mM glucose, 2 mM glutamine), or low-glucose and glutamine ↓Glc, Q (0.5 mM glucose, 0.2 mM glutamine) conditions under normoxia, transfected with siRNA targeting *HIF2A* or scrambled siRNA as a negative control, with the addition of 100 µM CoCl_2_ for protein detection. Gene expression (**A**) under standard conditions was assessed by PCR, and relative fold changes were determined after normalization to the PUM1 and HPRT1 housekeeping genes using the 2^−ΔΔCt^ method. Data are presented as box plots, with a horizontal line within the box representing the median and whiskers representing 1.5 × IQR per the Tukey method. Mann–Whitney test was used to compare the groups, with established statistical significance (α = 0.05, ns = not significant, ** *p* ≤ 0.001). A dashed line represents a baseline of 1.0 corresponding to the relative levels of reference proteins in cells treated with scrambled siRNA. HIF2 protein levels (**B**) were assessed by Western blot in the presence of 100 µM CoCl_2_, which served as a stabilization benchmark. Displayed bands are representative crops illustrating the average observed trend. Supplementary Materials provide full immunodetection images and a description of the statistical analysis, including the sample sizes and tests used.

**Figure 4:**
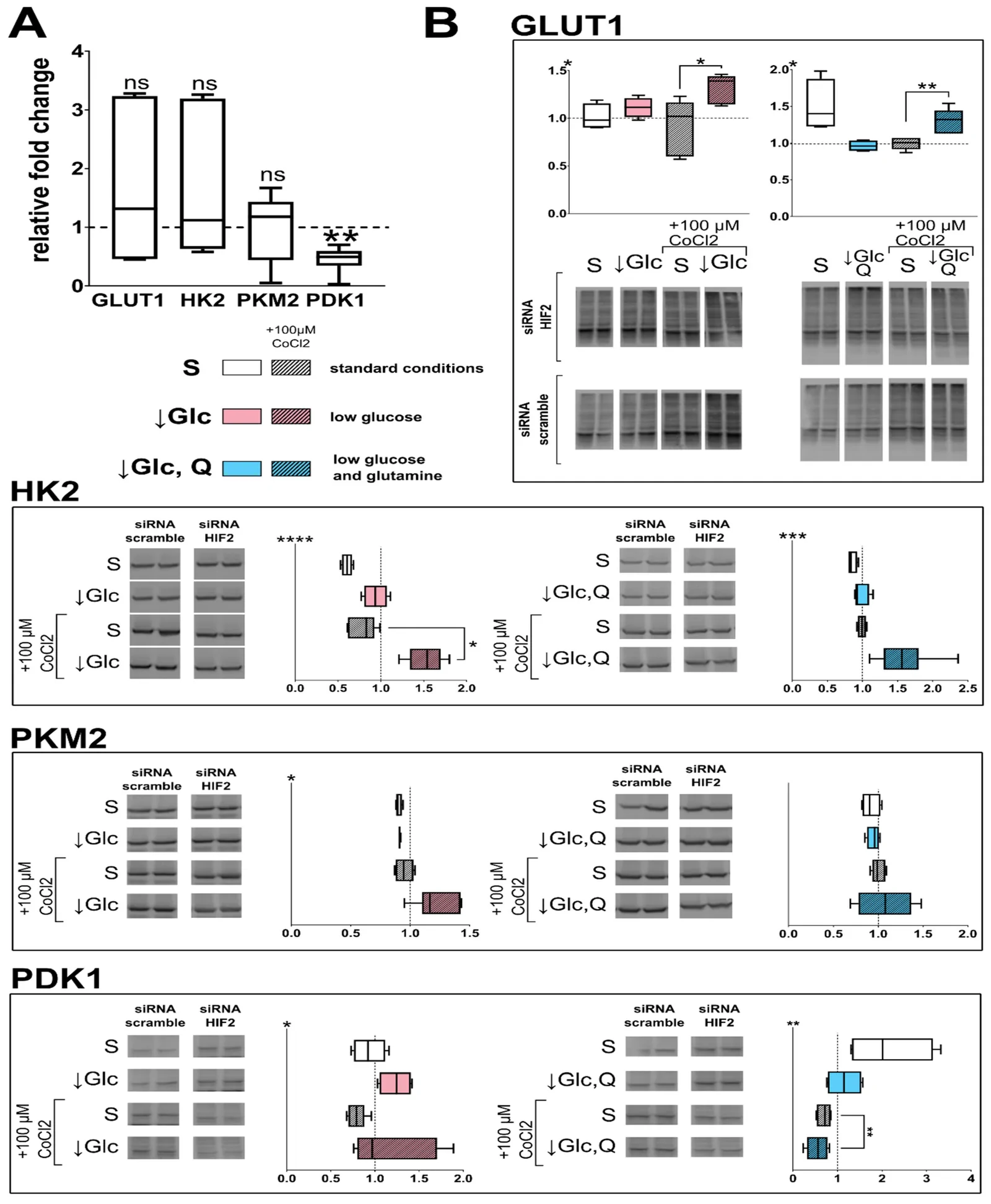
The effects of *HIF2A* silencing under different glucose and glutamine availability on the levels of proteins associated with metabolism. MDA-MB-231 cells were cultured for 48 h in standard S (25 mM glucose, 2 mM glutamine), low-glucose ↓Glc (0.5 mM glucose, 2 mM glutamine), or low-glucose and glutamine ↓Glc, Q (0.5 mM glucose, 0.2 mM glutamine) conditions under normoxia, transfected with siRNA targeting *HIF2A* or scrambled siRNA as a negative control, with the addition of 100 µM CoCl_2_. Gene expression (**A**) under standard conditions was assessed by PCR, and relative fold changes were determined after normalization to the PUM1 and HPRT1 housekeeping genes using the 2^−ΔΔCt^ method. The levels of GLUT1, HK2, PDK1, and PKM2 proteins were assessed by Western blot (**B**) and quantified by densitometry. Data are presented as box plots, with a horizontal line within the box representing the median and whiskers representing 1.5 × IQR per the Tukey method. Mann–Whitney test was used to compare the groups, with established statistical significance (α = 0.05, ns = not significant, ** *p* ≤ 0.001). A dashed line represents a baseline of 1.0 corresponding to the relative levels of reference proteins in cells treated with scrambled siRNA under matching conditions. Statistical analysis was performed using the Kruskal–Wallis test (indicated by the asterisks above the Y-axis of the respective protein graph), followed by Dunn’s post hoc test for multiple comparisons (indicated by asterisks above or adjacent to brackets connecting specific groups). Established statistical significance (α = 0.05, * *p* ≤ 0.01, ** *p* ≤ 0.001, *** *p* ≤ 0.0001, **** *p* ≤ 0.00001) is shown only for cells cultured under the same conditions regarding cobalt chloride addition. Displayed bands are representative crops illustrating the average observed trend. All quantitative observations were derived from the complete set of replicates. Supplementary Materials provide full immunodetection images and a description of the statistical analysis, including the sample sizes and tests used.

**Figure 5:**
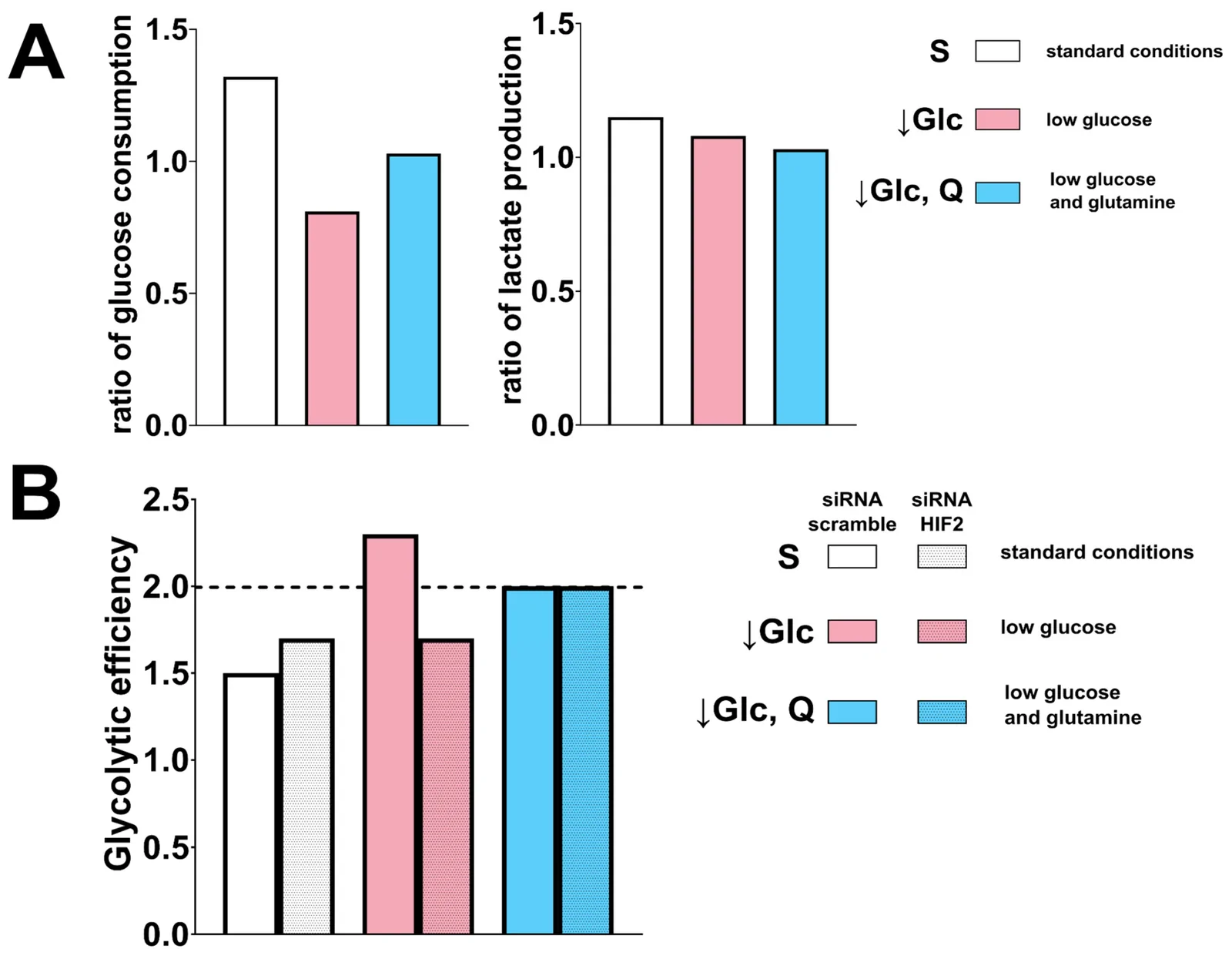
The effects of *HIF2A* silencing and glucose and glutamine availability on glucose consumption, lactate production, and glycolytic efficiency. MDA-MB-231 cells were cultured for 24 h in standard (25 mM glucose, 2 mM glutamine), low-glucose (0.5 mM glucose, 2 mM glutamine), or low-glucose and glutamine (0.5 mM glucose, 0.2 mM glutamine) conditions under normoxia, and transfected with siRNA targeting *HIF2A* or scrambled siRNA as a negative control. (**A**) Relative changes in glucose consumption and lactate production at 24 h. The bars show glucose consumption and lactate production (µmol per mg of protein mass) in cell culture media samples, expressed as the ratio of *HIF2A*-silenced cells to scrambled control. (**B**) Glycolytic efficiency, calculated as the stoichiometric ratio of lactate production to glucose consumption, at 24 h. Solid bars represent control cells treated with scrambled siRNA, and dotted bars represent HIF2-downregulated cells. The dashed horizontal line at 2.0 indicates the theoretical stoichiometric maximum of glycolytic efficiency.

## Data Availability

The data supporting the findings are provided within the article and its Supplementary Materials.

## Supplementary Materials

The following supporting information can be downloaded at: https://www.mdpi.com/article/10.3390/ijms27209306/s1.

## Abbreviations

The following abbreviations are used in this manuscript:

HIF	Hypoxia-inducible factor
GLUT1	Glucose Transporter Type 1
SLC2A1	Solute Carrier Family 2 Member 1
HK2	Hexokinase 2
PKM2	Pyruvate kinase M2
PDK1	Pyruvate dehydrogenase kinase 1
ARNT	Aryl Hydrocarbon Receptor Nuclear Translocator
PHDs	Prolyl hydroxylases (2-oxoglutarate-dependent dioxygenases)
OXPHOS	Oxidative phosphorylation
Akt	Protein Kinase B
DMEM	Dulbecco’s Modified Eagle Medium
FBS	Fetal bovine serum
siRNA	Small interfering RNA
TAE	Tris-acetate-EDTA
HPRT1	Hypoxanthine Phosphoribosyltransferase 1
PUM1	Pumilio 1
RIPA	Radioimmunoprecipitation assay
EDTA	Ethylenediaminetetraacetic acid
PMSF	Phenylmethylsulfonyl fluoride
PVDF	Polyvinylidene difluoride
TUBB3	Tubulin beta 3 class III
ROUT	Robust regression and outlier removal
TNBC	Triple-negative breast cancer
HBP	Hexosamine biosynthetic pathway
UDP	Uridine diphosphate (from UDP-glucose)
PFK1:	Phosphofructokinase-1
HIFAL	HIF antisense long non-coding RNA
LDH	Lactate dehydrogenase
PPP	Pentose Phosphate Pathway
ROS	Reactive oxygen species
