# Supplementary material for: Non-Canonical HIF2 Accumulation in Breast Cancer Cells
Source: Int J Mol Sci. 2026 Oct 20;27(20):9306. doi: 10.3390/ijms27209306 (PMC13705551; doi:10.3390/ijms27209306)
Supplement: Supplementary file 1 [file ijms-27-09306-s001.zip › List of antibodies.pdf]

Antibody	Type	Source	Product #	Dilution
HIF-1α (D2U3T) Rabbit mAb	Primary	Cell Signaling Technology	14179	1:2000
HIF-2α (E8E5Z) Rabbit mAb	Primary	Cell Signaling Technology	71565	1:2000
Glut1 (E4S6I) Rabbit mAb	Primary	Cell Signaling Technology	73015	1:2000
PDK1 (D37A7) Rabbit mAb	Primary	Cell Signaling Technology	5662	1:2000
PKM2 (D78A4) XP® Rabbit mAb	Primary	Cell Signaling Technology	4053	1:2000
Hexokinase II (C64G5) Rabbit mAb	Primary	Cell Signaling Technology	2867	1:2000
Pumilio 1 Antibody	Primary	Cell Signaling Technology	12322	1:2000
Anti-TUBB3 antibody (STJ97131)	Primary	St John's Laboratory	-	1:2000
Anti-rabbit IgG, HRP-linked Antibody	Secondary	Cell Signaling Technology	7074	1:5000
