# Supplementary material for: Non-Canonical HIF2 Accumulation in Breast Cancer Cells
Source: Int J Mol Sci. 2026 Oct 20;27(20):9306. doi: 10.3390/ijms27209306 (PMC13705551; doi:10.3390/ijms27209306)
Supplement: Supplementary file 1 [file ijms-27-09306-s001.zip › PCR/Primers/PCR primers sequences.docx]

| *HIF2A* | F: CCTTCCGACTCCCAGCATTC  R: GAGGCTGTCAGACCCGAAAA |
| --- | --- |
| *ncOGT* | F: CAGGAAGGCTATTGCTGAGAGG R: CGGAACTCACATATCCTACACGC |
| *HPRT1* | F: CATTATGCTGAGGATTTGGAAAGG R: CTTGAGCACACAGAGGGCTACA |
| *HPRT1a* | F: CCCTGGCGTCGTGATTAGTG  R: CACCCTTTCCAAATCCTCAGC |
| *PUM1* | F: GCATTTGGACAAGGTCTGGCAG R: GCTACAAGTCGAACAGGAGCTC |
| *GLUT1* | F: TTGCAGGCTTCTCCAACTGGAC  R: CAGAACCAGGAGCACAGTGAAG |
| *GLUT1*  *alternative* | F: CTGGCATCAACGCTGTCTTC  R: GCCTATGAGGTGCAGGGTC |
| *HK2* | F: GAGTTTGACCTGGATGTGGTTGC  R: CCTCCATGTAGCAGGCATTGCT |
| *HK2*  *alternative* | F: CAGTGGCAAAGGTGCAGC  R: TGCTCAGACCTCGCTCCATT |
| *PDK1* | F: CATGTCACGCTGGGTAATGAGG  R: CTCAACACGAGGTCTTGGTGCA |
| *PKM2* | F: ATGGCTGACACATTCCTGGAGC  R: CCTTCAACGTCTCCACTGATCG |
| *PKM2*  *alternative* | F: CGTGGATGATGGGCTTATTTCTC  R: TGCCTTGCGGATGAATGACG |

Sequences indicated by the red font color were excluded from subsequent experiments after evaluation of primers.
